# Incidence of Recurrent High-Grade Anal Dysplasia in HIV-1-Infected Men and Women Following Infrared Coagulation Ablation: A Retrospective Cohort Study

**DOI:** 10.3390/pathogens10020208

**Published:** 2021-02-14

**Authors:** Javier Corral, David Parés, Francesc García-Cuyás, Boris Revollo, Ana Chamorro, Carla Lecumberri, Antoni Tarrats, Eva Castella, Marta Piñol, Bonaventura Clotet, Sebastià Videla, Guillem Sirera

**Affiliations:** 1 Department of General Surgery, Hospital Germans Trias i Pujol, Carretera de Canyet, s/n, Badalona, 08916 Barcelona, Spain; dapares@gmail.com (D.P.); 31557fgc@gmail.com (F.G.-C.); 19483mpp@gmail.com (M.P.); 2 Lluita contra la Sida Foundation, Hospital Universitari Germans Trias i Pujol, Carretera de Canyet, s/n, Badalona, 08916 Barcelona, Spain; brevollo@flsida.org (B.R.); achamorro@flsida.org (A.C.); carlalecu@gmail.com (C.L.); atarrats.germanstrias@gencat.cat (A.T.); BClotet@irsicaixa.es (B.C.); gsirera@flsida.org (G.S.); 3 School of Medicine, Universitat Autónoma de Barcelona (UAB), Edifici M, Av. de Can Domènech, Bellaterra, 08193 Barcelona, Spain; 4 HIV Clinical Unit, Department of Medicine, University Hospital Germans Trias i Pujol, Carretera de Canyet, s/n, Badalona, 08916 Barcelona, Spain; 5 Department of Obstetrics and Gynaecology, Hospital Germans Trias i Pujol, Carretera de Canyet, s/n, Badalona, 08916 Barcelona, Spain; 6 Department of Pathology, Hospital Germans Trias i Pujol, Carretera de Canyet, s/n, Badalona, 08916 Barcelona, Spain; castella61@gmail.com; 7 Retrovirology Laboratory IrsiCaixa Foundation, Carretera de Canyet, s/n, Badalona, 08916 Barcelona, Spain; 8 Department of Clinical Pharmacology, Bellvitge University Hospital/IDIBELL/University of Barcelona, Hospitalet de Llobregat, Gran Via de les Corts Catalanes 199–203, 08907 Barcelona, Spain

**Keywords:** anal dysplasia, infrared coagulation, high-resolution anoscopy, HIV infection, anal intraepithelial neoplasia

## Abstract

This single-center, retrospective cohort study sought to estimate the cumulative incidence in HIV-1-infected patients of biopsy-proven high-grade anal intraepithelial neoplasia (HGAIN) recurrence after infrared coagulation (IRC) treatment. The study was based on data from a prospectively compiled database of 665 HIV-1-infected outpatients who attended a hospital Clinical Proctology/HIV Unit between January 2012 and December 2015. Patient records were checked to see which ones had received IRC treatment but later experienced a recurrence of HGAIN. Cytology samples were also checked for the presence of human papilloma virus (HPV). A total of 81 of the 665 patients (12%, 95%CI: 10–15%), of whom 65 were men and 16 women, were diagnosed with HGAIN and again treated with IRC. Of these 81, 20 (25%) experienced recurrent HGAIN, this incidence being true of both men (16/65, 95%CI: 19–57%) and women (4/16, 95%CI: 10–50%). The median time to recurrence was 6 (2–19) months overall, 6 (2–19) months in men, and 4 (2–6) months in women. HPV infection was detected in all patients except two, with HPV-16 being the most common genotype. This rate of incidence of recurrent HGAIN following IRC treatment is consistent with other reports and highlights the importance of continued post-treatment surveillance, particularly in the first year.

## 1. Introduction

Anal cancer (AC) is one of the most common non-AIDS-defining cancers [1], and its incidence has increased in recent decades. Many studies have pointed in particular to its rise among HIV-1-infected men, especially men who have sex with other men (MSM). However, data on AC incidence among HIV-1-infected women are scarce.

Interrupting the natural history of AC is a challenge. Infrared coagulation (IRC) has been established as an effective treatment for high-grade squamous intraepithelial lesions (HSIL) [2]. It can be applied at the doctor’s office with local anesthesia and shows a low rate of complications after treatment. First, the anal canal lesion is identified by means of high-resolution anoscopy (HRA), then IRC is applied directly using a short pulse of narrow-beam light, which produces thermal coagulation necrosis [3]. However, even after IRC, anal intraepithelial neoplasia (AIN) may appear. High-grade AIN (HGAIN, which incorporates AIN grades 2 and 3) is often a precursor of AC.

In a previous study [4], our group reported a cumulative incidence of biopsy-proven HGAIN recurrence after IRC treatment in 56 HIV-1-infected patients of 12.5% (95%CI: 6–24%); broken down by gender, recurrence was seen in two out of 11 women (18%) and five out of 45 men (11%). Having accumulated a larger body of data which also incorporates a three-year follow-up period, in the present study we will expand on our earlier results.

## 2. Patients and Methods

### 2.1. Study Design

The study was a single-center, retrospective cohort study based on data from a prospectively compiled database consisting of the digitized medical records of outpatients who were being attended at the HIV Unit of the Hospital Germans Trias i Pujol in Badalona, Spain. The cohort protocol was approved by the local Institutional Review Board (IRB) and all patients gave their written informed consent.

### 2.2. Study Population

The study population was made up of the 665 HIV-1-infected patients of both genders who had been treated using IRC for biopsy-proven HGAIN between 1 January 2012 and 31 December 2015 and who had yielded positive HIV serology results, were being attended at the hospital’s HIV Unit, and were at least 18 years old. Follow-up of all these patients had ceased by December 2019.

### 2.3. Anal Canal: Cytological and Histological Assessment

An anal canal sample was taken for cytological examination and the Papanicolaou test was performed to check for anal canal cytological changes, as described previously [4].

After topical application of 5% acetic acid in the anal canal for 2 min, HRA was performed using the technique described in [5]. If the HRA revealed a lesion, a biopsy was performed. Histological changes were classified according to the grade of AIN as AIN-1, AIN-2, or AIN-3.

### 2.4. Infrared Coagulation

If the result of the histological examination revealed HGAIN (i.e., AIN-2 or AIN-3), patient consent to undergo IRC ablation was obtained. IRC sessions were usually scheduled between 1 and 2 months after the first HRA. During the IRC process, each lesion was identified, infiltrated with local anesthesia, and then repeatedly coagulated using a Redfield IRC 2100 Infrared Coagulator (Redfield Corporation, Rochelle Park, New Jersey, USA) in pulses of 1.5 s [4]. All procedures were performed by trained surgeons.

### 2.5. Follow-up after IRC Ablation

After IRC ablation, patients underwent routine evaluations involving visual inspection, digital rectal examination, and anal canal cytology at 3 to 6-month intervals. Major post-IRC complications were recorded. Patients with abnormal cytology results during the follow-up underwent another examination with HRA. If HRA revealed a new lesion, a biopsy was again performed. Patients with biopsy-proven AIN-2 or AIN-3 were advised to undergo further IRC ablation. Patients with AIN-1 were monitored with anal cytology at 3 to 6-month intervals. Patients with normal cytology results after IRC were monitored using anal cytology at 12-month intervals.

### 2.6. Cervix: Cytological Assessment

In the group of women studied, cervical cytology was assessed (in the same year as the anal canal IRC ablation) using the Papanicolaou method and results recorded according to the Bethesda Classification System, as follows: normal (i.e., negative for intraepithelial lesion or malignancy); ASCUS (atypical squamous cells of undetermined significance); LSIL (low-grade squamous intraepithelial lesions); or HSIL.

### 2.7. Detection of Human Papilloma Virus Infection

When samples were available from the anal canal or, in the case of women, the cervix, these samples were tested for human papilloma virus (HPV) infection with positive results simultaneously identified for HPV type using the Anyplex^TM^ II HPV28 real-time PCR assay (Seegene, Seoul, Korea). DNA was extracted from cell suspensions using the QiAMP Viral RNA kit (QIAGEN, Hilden, Germany).

### 2.8. Study Definitions

Baseline was defined as the moment of the patient’s first anal cytology assessment at which HGAIN was recorded, with recurrent HGAIN defined as biopsy-proven HGAIN subsequent to IRC treatment. The follow-up period was defined as the time between baseline and either biopsy-proven recurrence of HGAIN or last visit available after IRC in case of no recurrence.

### 2.9. Statistical Analysis

No formal sample size was calculated. The sample was defined as all HIV-1-infected patients with HGAIN (AIN-2 or AIN-3) as diagnosed by histological examination.

The cumulative incidence of biopsy-proven HGAIN recurrence after IRC treatment was estimated, and its 95% confidence interval (95%CI) was calculated. The mean time to recurrence was analyzed using the Kaplan–Meier method. Agreement between the cytological diagnosis (Papanicolaou test) and the histological diagnosis (biopsy) was described. HPV infection and IRC safety was described.

A *p* value <0.05 was considered statistically significant. Data were analyzed using SPSS version 20 statistical software (SPSS, Inc., Chicago, IL, USA).

## 3. Results

### 3.1. Patient Characteristics

Of the 665 HIV-1-infected patients who were attended at our Clinical Proctology HIV Section with at least an anal canal cytology performed, 81 (12%, 95%CI: 10–15%) patients were diagnosed with HGAIN and were treated with IRC. Of these 81, 65 (80%) were men, of whom 60 (92%) were Men who have Sex with Men (MSM) and 5 (8%) were Men who have Sex with Women (MSW), and 16 (20%) were women. The mean age was 43.1 (SD 11.2) years and the median 44 (range 24–77 years). Table 1 shows the baseline characteristic of the study population.

### 3.2. Recurrence of HGAIN

The cumulative incidence of recurring biopsy-proven HGAIN was 25% (20/81, 95%CI: 17–35%): 25% (16/65, 95%CI: 19–57%) in men and 25% (4/16, 95%CI: 10–50%) in women. The median follow-up period for the study population was 55 months (range 33–71) and the median time to recurrence was 6 months (range 2–19). Broken down by gender, the median was 6 months (range 2–19) in men and 4 months (range 2–6) in women.

At baseline, a total of 101 anal lesions in the 81 patients were visually detected by HRA: 30% (19/64) of the men had >1 lesion whereas all the women had only one lesion. At recurrence, 26 lesions in the 20 patients were detected by HRA; here, 6% (1/16) of the men and 50% (2/4) of the women had >1 lesion.

### 3.3. Cytology Results at Anal Canal and Cervical Sites at Baseline

Agreement between the cytological and histological diagnosis was described (Table 2). All patients (*n* = 81) with abnormal cytological diagnosis (ASCUS, LSIL or HSIL) and visualized lesion by HRA had a histological diagnosis of AIN-2 (61 patients, 75%, 95%CI: 65–83%) or AIN-3 (20 patients, 25%, 95%CI: 17–35%).

The baseline anal canal cytology test results for the men (*n* = 65) were ASCUS 8% (5/65), LSIL 59% (38/65), and HSIL 34% (22/65), while, for the women (*n* = 16), they were ASCUS 6% (1/16), LSIL 31% (5/16), and HSIL 63% (10/16). The baseline histology results for the men show that 79% (51/65) had AIN 2 and 22% (14/65) had AIN 3, while 63% of the women (10/16) had AIN 2 and 38% (6/16) had AIN 3.

At recurrence, the anal canal cytology test results for the men (*n* = 16) were ASCUS 6% (1/16), LSIL 31% (5/16) and HSIL 63% (10/16), while, for the women (*n* = 4), they were LSIL 25% (1/4) and HSIL 75% (3/4). The recurrence histology results for the men show that 63% (10/16) had AIN 2 and 38% (6/16) had AIN 3, while 50% of the women (2/4) had AIN 2 and 50% (2/4) had AIN 3.

With regard to cervical site testing, cytology was available for all the female participants with HGAIN in the anal canal. Cytology test results in this case showed ASCUS in 12% of the women (2/16) and LSIL in 38% (6/16), the remaining 50% women (8/16) yielding normal (negative) results. The four women who presented a recurrence of HGAIN at an anal canal site had at least one historical cervical dysplasia, as revealed in the baseline cytology test.

### 3.4. HPV Infection

At baseline, 67 out of 81 patients (83%; 12 women, 55 men) had samples from an anal canal site for available HPV infection detection. HPV infection was detected in all patients except two (one woman, one man). Single HPV infection (for only one HPV genotype) was detected in five patients (four women, one man), and multiple infections (for >1 HPV genotype) in 60 patients (seven women, 53 men). HPV-16 was the most prevalent HPV genotype found at an anal canal site at baseline; this represented 40% of the men (22/55, 95%CI: 29–54%) and 50% of the women (6/12, 95%CI: 25–75%). Men presented a greater number of HPV genotypes at anal canal sites (mean 6.1, SD 3.1) relative to women (mean 3.8, SD 2.7).

Of the 20 patients with recurrent HGAIN, 18 (90%; three women, 15 men) had samples from an anal canal site available for the detection of HPV infection. The analysis detected 28 HPV genotypes, of which 13 were high-risk-HPV genotypes (16, 18, 31, 33, 35, 39, 45, 51, 52, 56, 58, 59, and 68), 9 were low-risk-HPV genotypes (6, 11, 40, 42, 43, 44, 54, 61, and 70) and 6 were uncertain-risk-HPV genotypes (26, 53, 66, 69, 73, and 82). HPV infection involving >1 HPV genotype was detected in all patients. HPV-16 was the most prevalent HPV genotype, being present in 47% of the men (95%CI: 25–70%) and 100% of the women (95%CI: 44–100%). The men presented a greater number of HPV genotypes at anal canal sites (mean 4.7, SD 1.5) relative to the women (mean 3.9, SD 2.3).

### 3.5. Adverse Events Related to IRC

No major complications related to IRC were reported. Pain was the main adverse event reported by patients. Likewise, no adverse events related with the obtaining of sampled from the anal canal for cytology and HPV assessment were reported.

## 4. Discussion

HGAIN is a prevalent pathology among HIV-1-infected patients, and recurrence of HGAIN in these patients, regardless of treatment, is not uncommon. In the present study of 81 HIV-1-infected patients with a long follow-up (minimum 33 months), we found the same cumulative incidence of biopsy-proven HGAIN recurrence after IRC treatment in both men and women, namely 25%.

Though our previous results (published in 2013) on the estimated cumulative recurrence of biopsy-proven HGAIN seem to differ from those reported here, the confidence intervals overlap (12.5%, 95%CI: 6–24% versus 25%, 95%CI: 17–35%) [6]. Furthermore, the rates of HGAIN recurrence reported here are similar to those reported by most other authors, which range from 12.5% to 38% [4,6,7,8,9,10], though a recent study reported a recurrence rate of 53.3% at 2 years in a cohort of 100 MSM [11] (it is noteworthy that these studies were mainly based on MSM).

Our study has several limitations. First, the small sample size (especially of women) means that the results found may be an over- or underrepresentation of the real recurrence rate. Second, our study reports the experience of a single center where a screening program for anal cancer prevention has been active since 2005. However, this program may differ from others [12], which may limit the applicability of our results to other centers. Third, it is conceivable that some cases of HGAIN were missed as a result of the fact that the first step in our screening program is performed by means of cytological analysis. Finally, although we report here the number of lesions detected by means of HRA, we do not stipulate the actual size of every lesion. On the other hand, our study also has strengths. To our knowledge, it is the first study to include HIV-1-infected women and data concerning their gynaecological history and HIV/HPV infection. The differences between HIV-1-infected men and women in the natural history of HPV-infection in the anal canal are not well understood. Despite the low number of women studied, the rate findings of HGAIN, of AIN-3 and of HPV 16 infection at anal canal were greater among HIV-1-infected women than HIV-1-infected men (at both moments studied: baseline and recurrence). Therefore, these results may suggest that HPV infection could be more aggressive among HIV-1-infected women and, in consequence, HIV-1-infected women could have a greater risk of HGAIN recurrence after IRC treatment.

In conclusion, the fact that recurrence of HGAIN in HIV-1-infected patients after IRC ablation is a fairly common occurrence highlights the great importance of continued post-treatment surveillance to prevent new episodes, particularly in the first year.

## Figures and Tables

**Table 1:** Baseline characteristics.

Baseline Characteristics	Study Population N = 81	Women *n* = 16	Men *n* = 65	*p*-Value
Age in years				
Median (Range *)	44 (24–77)	46.5 (24–59)	43 (24–77)	0.425
Time of known HIV in years				
Median (Range *)	5 (0–30)	23 (5–30)	4 (0–30)	<0.001
Antiretroviral therapy				
Yes (%)	73/81 (90%)	15/16 (94%)	58/65 (89%)	0.503
HIV plasma load in copies/mL				
Mean at zenith (SD)	142 064 (243 485)	59 063 (79 322)	162 495 (265 490)	0.129
Current mean (SD)	7 1250 (22 050)	5 343 (18 381)	7 565 (22 970)	0.72
N patients < 50 HIV RNA (%)	62/81 (76.5%)	11/16 (68.8%)	51/65 (78.5%)	0.302
CD4 cell count/uL				
Current mean (SD)	572 (250)	482 (252)	594 (247)	0.108
Mean at nadir (SD)	246 (170)	182 (159)	261 (169)	0.096
N patients < 200 cells/uL at nadir (%)	35/81 (43%)	10/16 (63%)	25/65 (39%)	0.073
Hepatitis C				
Yes (%)	20/81 (24.7%)	10/16 (62.5 %)	10/65 (15.4%)	<0.001

* Range (minimum-maximum values).

**Table 2:** Cytological and histological diagnosis at the anal canal.

Cytology-Histology Concordance	Histology Results (Biopsy)	
	AIN-2	AIN-3
Baseline cytology results (*n* = 81)		
ASCUS (*n* = 6)	5 (83%)	1 (17%)
L-SIL (*n* = 43)	32 (74%)	11 (26%)
H-SIL (*n* = 32)	24 (75%)	8 (25%)
At recurrence cytology results (*n* = 20)		
ASCUS (*n* = 1)	1 (100%)	
L-SIL (*n* = 6)	4 (67%)	2 (33%)
H-SIL (*n* = 13)	7 (54%)	6 (46%)

## Data Availability

All raw data is available and provided upon request.
